# Detection of the *ABCB1*1930_1931del TC Mutation in Two Suspected Ivermectin-Sensitive Cats and Their Relatives by a Novel TaqMan Allelic Discrimination Assay

**DOI:** 10.3389/fvets.2021.808392

**Published:** 2022-02-21

**Authors:** Daniela Nürnberger, Lisa Wagner, Simon F. Müller, Silke Leiting, Regina Leidolf, Jörg Alber, Melanie Hamann, Joachim Geyer

**Affiliations:** Faculty of Veterinary Medicine, Institute of Pharmacology and Toxicology, Justus Liebig University Giessen, Giessen, Germany

**Keywords:** P-glycoprotein, MDR1, ABCB1, ivermectin-sensitivity, adverse drug reaction, allelic discrimination, TaqMan, cat

## Abstract

The multidrug resistance gene MDR1 (syn. ABCB1) encodes for the multidrug efflux transporter P-glycoprotein (P-gp), which is highly expressed at the blood-brain barrier and protects the brain from potentially neurotoxic compounds, such as ivermectin. MDR1 mutation in dogs is known to be linked to dramatically increased brain accumulation of ivermectin and life-threatening neurological toxicity. The present report describes two suspected ivermectin-sensitive Maine Coon cats, which exhibited neurological toxicity following subcutaneous application of therapeutic doses of ivermectin. Both cats showed a homozygous 2-bp deletion in the MDR1/ABCB1 coding sequence (*ABCB1*1930_1931del TC, syn. MDR1 nt1930(del2)) that had previously been associated with a drug-sensitive phenotype in cats. For cat MDR1 genotyping, a novel TaqMan allelic discrimination assay was established and validated. This assay was used for *ABCB1*1930_1931del TC genotyping of the drug-sensitive cats as well as of more than 50 relatives. About half of them had the heterozygous MDR1(+/-) genotype, while none of these related cats with former ivermectin treatment had a history of drug-sensitivity. In conclusion: The present study supports previous findings on drug-sensitivity in cats with homozygous *ABCB1*1930_1931del TC mutation. The newly established TaqMan allelic discrimination assay provides a useful and reliable method for routine MDR1 genotyping in cats in order to identify drug-sensitive cats prior to treatment with established P-gp substrates such as ivermectin and other macrocyclic lactones and thus to improve therapeutic safety.

## Introduction

The multidrug-resistance gene MDR1 (syn. ABCB1) encodes for P-glycoprotein (P-gp), a multidrug efflux transporter from the ATP-binding cassette (ABC) transporter family, which is expressed in many different mammalian tissues (1). In the blood-brain barrier, P-gp restricts the entry of drugs and toxins into the central nervous system (CNS) and so protects the brain from potentially neurotoxic compounds (2).

Genetic variations in the MDR1 gene can lead to abolished P-gp drug efflux function and have been associated with increased drug susceptibility due to drug accumulation in the CNS (3). Almost 20 years ago, Mealey et al. (4) identified a 4-bp gene deletion mutation (MDR1-1Δ, syn. MDR1 nt230(del4), syn. ABCB1-1Δ) in the canine MDR1 gene (4). This mutation is associated with the ivermectin-sensitive phenotype, which was first observed in Collie dogs in the early 1980s (so called *ivermectin-sensitive Collies*) (5). Subsequent genotyping studies revealed that many other dog breeds are predisposed to this MDR1 mutation, such as Border Collies, Australian Shepherds, Shetland Sheepdogs, Longhaired Whippets, and White Shepherds (6–9). In addition to ivermectin, dogs with homozygous nt230(del4) MDR1 mutation showed increased drug-sensitivity to some other P-gp substrates commonly used in veterinary medicine, including e.g. the macrocyclic lactones doramectin and moxidectin, or emodepside (10, 11). In these cases, neurological toxicity included ataxia, mydriasis, temporary blindness, hypersalivation, tremors, lethargy, coma, and sometimes even death (12).

Recently, a suspected loss-of-function MDR1 mutation was also identified in a small number of cats, which exhibited neurological toxicity when exposed to ivermectin or eprinomectin similar to that of MDR1 mutant dogs (13, 14). This 2-bp deletion in the MDR1 coding sequence was referred to as *ABCB1*1930_1931del TC (13, 14).

Here, we report on two further cats (*Felis catus*) exhibiting neurological toxicity after ivermectin treatment. The objective of this study was to determine whether these cats (one Maine Coon and one Maine Coon mix) were affected by this previously described 2-bp deletion mutation (syn. MDR1 nt1930(del2)) in the feline MDR1 gene as well. In addition, the MDR1 genes of more than 50 relatives were analyzed for this mutation. For this purpose, a TaqMan allelic discrimination (AD) method, which is already available for routine MDR1 genotyping in dogs (15), was established in the present study and was validated for use in cats.

## Materials and Methods

### Animals and DNA Samples

DNA sampling for this study was reviewed and registered by the local Animal Welfare Authorities (Regierungspräsidium Giessen; registration no: 19 c 20 15 h 02 Gi 18/11 kTV 10/2021). DNA was obtained from two cats (one female purebred Maine Coon and one male Main Coon mix) that developed neurological toxicity following exposure to ivermectin. All relevant data on these suspected adverse drug reactions were collected by contacting the animal owner following standard procedure for pharmacovigilance cases. Additional DNA samples were obtained from related cats (*n* = 52) and all were used for diagnostic MDR1 genotyping. For all cats, it was asked explicitly whether ivermectin had been used in the past and whether any signs of neurological toxicity subsequently appeared. Blood samples (1 ml of EDTA whole blood) and/or buccal swabs (two per animal) were received from all these cats and used for isolation of genomic DNA. In addition, surplus blood samples (*n* = 5) of cats were kindly provided by the local Clinic for Small Animals (Justus Liebig University Giessen, Germany) for validation of the TaqMan AD method. Genomic DNA was isolated from blood samples using Nucleo Spin Blood QuickPure Kit (Macherey-Nagel, Düren, Germany), or from buccal swabs using QIAamp DNA Mini Kit (Qiagen, Hilden, Germany).

### Detection of the Feline *ABCB1*1930_1931del TC Mutation by TaqMan AD

The TaqMan AD method (15, 16) was chosen for the detection of the feline *ABCB1*1930_1931del TC mutation. Briefly, a Custom TaqMan SNP Genotyping Assay (Applied Biosystems, Waltham, USA) was used. GenBank feline gDNA and cDNA sequences (Accession No. NC_018724.3 and NM_001171064.2, respectively) as well as cat MDR1 DNA reference sequences from our lab (GenBank Accession No. GU222365) served as templates for primer and probe design. All sequences of the gene-specific oligonucleotide primers and the fluorescent-labeled allele-specific oligonucleotide probes are listed in Table 1. The assay also included a minor groove binder (MGB) at the 3'end of each probe to increase differences in melting temperature (Tm) for more accurate AD.

**Table 1:** Primer and probe sequences used for cat MDR1 PCR amplification and genotyping.

**Forward primer (5' → 3')**	**Allele-specific probes (5' → 3')**	**Reverse primer (5' → 3')**	**Fragment (bp)**
ACA AGA GGA AAT GAA ATT GAA TTA GAA AAT GCA	VIC-CAA TTT CAC TTA TGG ATT CAT-NFQ *MDR1(+)*	CTG GAT CCT GAA TCT TTT GGA GAC A	93/95
	FAM-CAT CAA TTT CAC TTA TGT TCA T-NFQ *MDR1(-)*		
GGT CCA AAG CAA ATA GGA TTG		TGT CTC CAC AAA CAT TCA ACC	823

Real-time PCR amplification was performed in a total reaction volume of 10 μl, consisting of 5 μl TaqMan Genotyping Master Mix (2X) (Applied Biosystems, Waltham, USA), 0.5 μl TaqMan Assay Mix (20X working stock solution) and 4.5 μl of genomic DNA. The TaqMan Genotyping Master Mix contained the ROX reference dye for automated signal normalization as well as the AmpliTaq Gold DNA polymerase, dNTPs and reaction buffer. Samples were amplified and detected in a MicroAmp Fast 96-Well Reaction Plate (Applied Biosystems, Waltham, USA) on an Applied Biosystems StepOnePlus Real-Time PCR System. The following PCR protocol was used consisting of a pre-PCR plate read at 60°C for 30 sec, a holding stage at 95°C for 10 min followed by 40 cycles of 95°C x 15 sec and 60°C x 1 min and a post-PCR plate read at 60°C for 30 sec. StepOne Software 2.3 (Applied Biosystems, Waltham, USA) was used to analyze the automatically determined AD data.

### Evaluation of the TaqMan AD Method by DNA Sequencing

**Figure 1:**
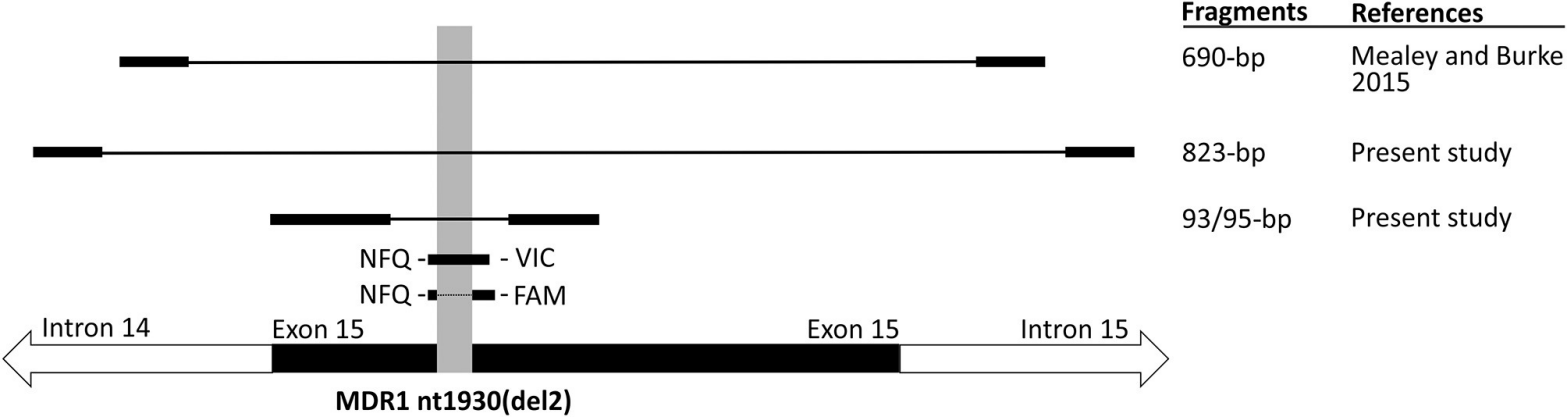
Schematic overview of the localization of the primer and probe sequences used for cat MDR1 genotyping. For comparison, localization of the primers used for cat MDR1 genotyping in the previous study by Mealey and Burke (13) is indicated. Localization of the *ABCB1*1930_1931del TC mutation (syn. MDR1 nt1930(del2)) is indicated by shading.

To validate the results of the TaqMan AD analysis, all genomic DNA samples were additionally verified by DNA sequencing. DNA sequences were also used to preclude any SNP at the sites of primer and probe hybridization, since this could affect accurate PCR amplification. For this purpose, PCR primers were designed (see Table 1) to generate 823-bp amplicons covering exon 15, including the site of *ABCB1*1930_1931del TC mutation and all TaqMan primer and probe binding sites (Figure 1). Primers were ordered by Metabion (Planegg, Germany). PCR amplification was performed with Phusion Flash High-Fidelity PCR Master Mix (Thermo Scientific, Waltham, USA). For each reaction, 1 μl of gDNA was used in a total reaction volume of 20 μl. For amplification, a touchdown protocol was chosen consisting of an initial denaturation at 98°C for 2 min, followed by 10 loops of touchdown cycling with denaturation (98°C for 15 s), annealing (61–0.5°C per cycle, 15 s) and elongation (72°C for 35 s). After this touchdown phase, 30 cycles with denaturation of 98°C for 15 s, annealing of 56°C for 15 s and elongation at 72°C for 35 s + 1 s per step was conducted, followed by a final elongation for 7 min at 72°C. From each PCR product, 5 μl were visualized on a 1.5 % agarose gel for control before the remaining 15 μl were purified using GeneJET PCR Purification Kit (Thermo Scientific, Waltham, USA). Subsequently, bi-directional Sanger sequencing was performed by Microsynth Seqlab (Göttingen, Germany) with the same primers used for amplification. All sequencing data were analyzed and aligned using Finch TV 1.4 and DNASTAR 16.0 software (Lasergene).

## Results

### Detection of the *ABCB1*1930_1931del TC in Two Suspected Ivermectin-Sensitive Maine Coon Cats by DNA Sequencing

In the present study, we report on two suspected ivermectin-sensitive cats. One of them (cat # 1), a female 5-year-old Maine Coon cat with ~5.5 kg BW, was treated with a subcutaneous injection of 0.2 mg/kg ivermectin and 0.1 ml/kg praziquantel (corresponding to the recommended therapeutic dose of 5.7 mg/kg praziquantel). The evening after treatment, the cat developed neurological signs that worsened in the first days, including mydriasis, temporary blindness, ataxia, tremor, apathy, depression, somnolence, increased sensitivity to light, touch and sound, and hypersalivation. Symptoms lasted for several weeks. According to the owner, the cat had developed similar neurological signs after receiving the same combination of ivermectin and praziquantel one year before.

The second cat (# 2), a 7-month-old male Maine Coon mix with approximately 6–7 kg BW, was also treated with a subcutaneous injection of 0.1 mg/kg ivermectin and 0.1 ml/kg praziquantel (corresponding to the recommended therapeutic dose of 5.7 mg/kg praziquantel). A few hours later, the cat developed similar neurological signs including mydriasis, ataxia, tremor, twitching, depression, somnolence and increased sensitivity to light, touch and sound. Symptoms lasted for several days and were less severe compared to cat # 1. Both cats belong to the same owner, but treatment was carried out independently.

DNA sequencing revealed that both suspected ivermectin-sensitive Maine Coon cats were homozygous for the *ABCB1*1930_1931del TC mutation, which had previously been described and associated with a drug-sensitive phenotype in cats (13, 14).

### Development and Validation of an *ABCB1*1930_1931del TC TaqMan AD Genotyping Method

**Figure 2:**
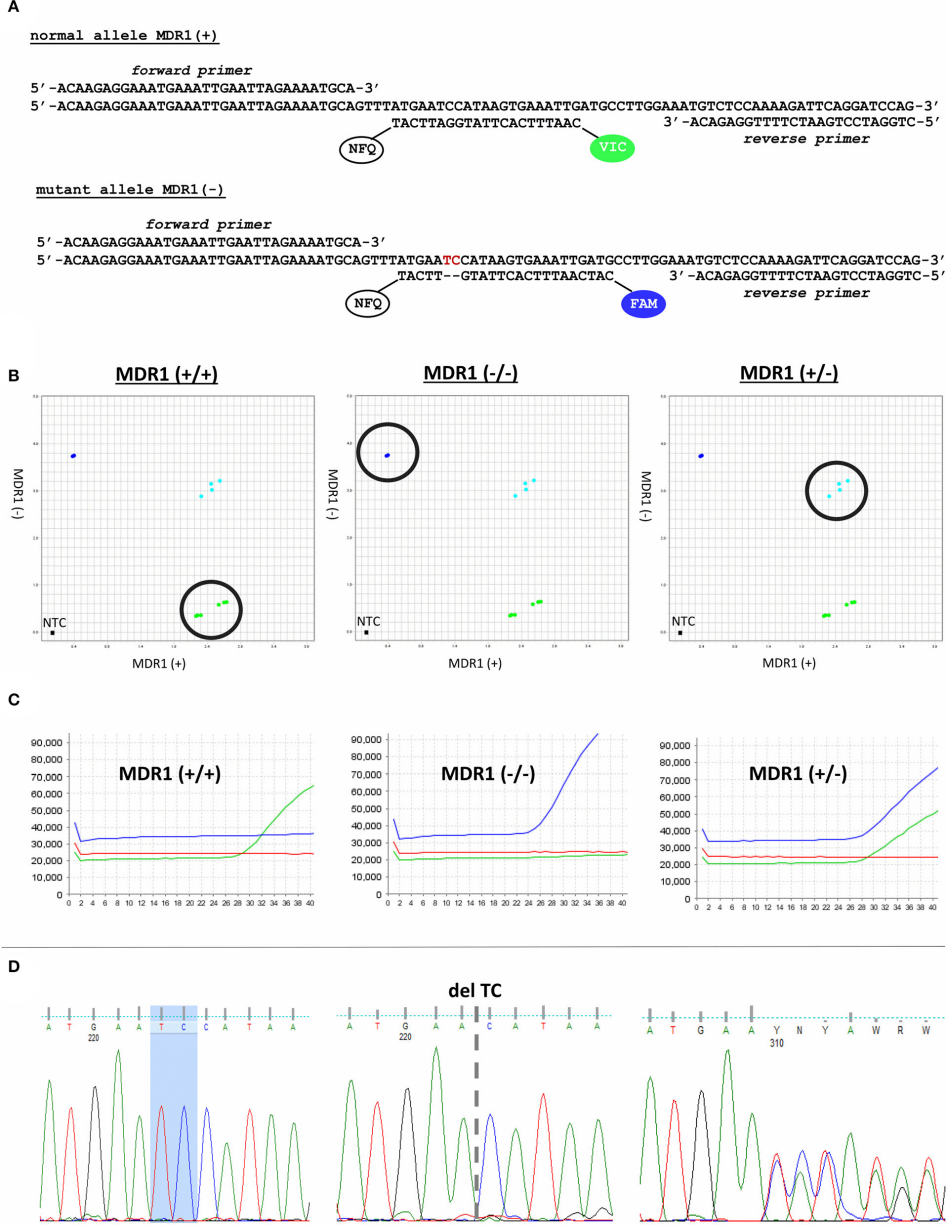
Cat MDR1 genotyping. **(A)** Gene-specific primers and allele-specific probes of the TaqMan AD assay. **(B)** AD plot revealed discrete clusters for all three genotypes. **(C)** Fluorescence readouts from the real-time PCR amplification revealed clear separation between fluorescence signals from the VIC-coupled wild-type probe and the FAM-coupled mutant probe. **(D)** Verification of the TaqMan AD genotyping calls by Sanger sequencing.

For cat MDR1 genotyping, a TaqMan AD assay with gene-specific primers and allele-specific probes was developed (Figure 2A). The AD plot showed the typical three discrete clusters and the no-template controls (NTC), for which samples without DNA were used (Figure 2B). The individual points in each cluster were grouped together and each cluster was clearly separated from the other two clusters, allowing reliable genotyping. In the fluorescent readout, samples homozygous for the wild-type allele [MDR1(+/+)] only showed increase of VIC fluorescence, while samples homozygous for the *ABCB1*1930_1931del TC mutant allele [MDR1(-/-)] only revealed increase for the FAM dye. Samples with heterozygous genotype [MDR1(+/-)] showed increasing fluorescence from both dyes (Figure 2C).

For validation of the TaqMan AD genotyping calls, results were confirmed by Sanger sequencing (Figure 2D), which is considered the gold-standard technology for verification of PCR results (17). Genotypes determined by the TaqMan AD assay generally matched with the obtained DNA sequences. Regarding the starting material, DNA isolated from blood was most appropriate for MDR1 genotyping and revealed 100% success rates, while DNA isolated from buccal swabs failed in ~10% of the cases, most probably due to insufficient amount and quality of DNA. However, in almost all of these cases the genotypes could be determined when using DNA from the second buccal swab. Only in three out of 54 animals sampled by buccal swab, it was not possible to assign an MDR1 genotype *via* TaqMan AD, but only by DNA sequencing. Apart from confirming the MDR1 genotype, DNA sequencing data were also used to identify any SNP at the sites of primer and probe hybridization of the TaqMan AD assay. There were no sequence variations at the primer and probe hybridization sites among the overall 59 samples analyzed.

### MDR1 Genotyping of 52 Relatives of the Two Ivermectin-Sensitive Cats

**Figure 3:**
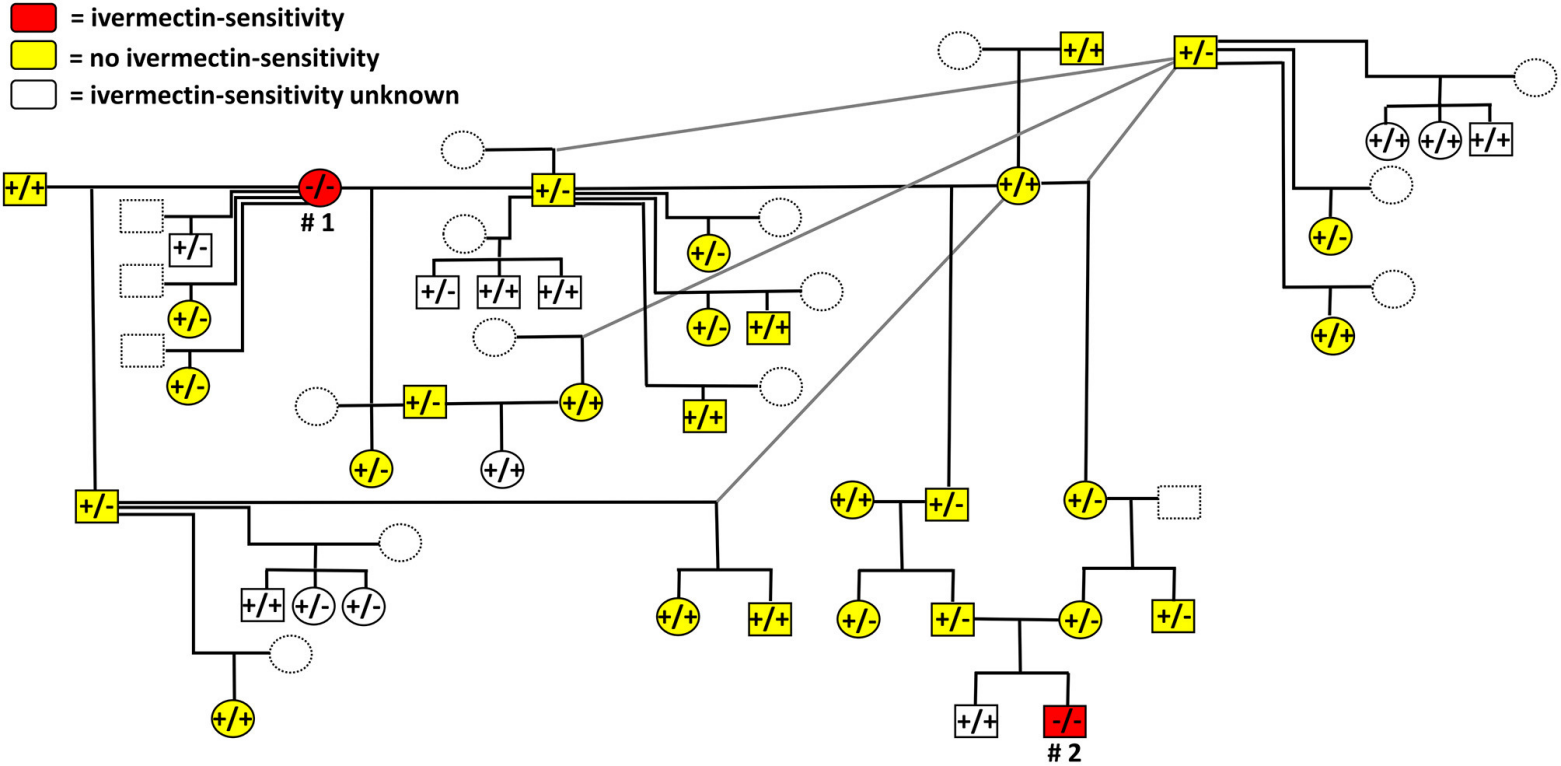
Schematic and exemplary illustration of the anonymized pedigree, MDR1 genotypes and drug-sensitive phenotypes of the cats analyzed in the present study. Documented drug-sensitivity is indicated by red color for the ivermectin-sensitive female (# 1) and male (# 2) Maine Coon cats. Cats without history of neurological toxicity after ivermectin treatments in the past are indicated in yellow. Within the pedigree, circles represent females and boxes represent males. Individual genotypes are indicated as follows: MDR1(+/+), homozygous wild-type; MDR1(+/-), heterozygous mutant or MDR1(-/-), homozygous mutant for the genotype *ABCB1*1930_1931del TC. Dotted lines were used for cats that were not sampled/genotyped. For better clarity, only a limited number of 41 out of the overall 54 related cats were included in this figure.

A total of 52 relatives of the two suspected ivermectin-sensitive Maine Coon cats were included in the MDR1 genotyping. None of them showed the homozygous MDR1(-/-) genotype, but 27 were heterozygous for the *ABCB1*1930_1931del TC mutation and 25 showed the wild-type genotype [MDR1(+/+)]. According to the owners, many of these related cats were also treated with ivermectin in the past, but none of them subsequently developed neurological signs. A schematic and exemplary overview of individual genotyping results together with the corresponding relationships as well as information on the ivermectin-sensitive phenotype are shown within an anonymized pedigree in Figure 3. For better clarity, only a limited number of 41 out of the overall 54 related cats were included in this figure.

## Discussion

The present study analyzed two suspected ivermectin-sensitive Maine Coon cats by a newly developed MDR1 genotyping method for cats and found homozygous *ABCB1*1930_1931del TC mutation in both animals. This strongly suggests a causal relationship to the observed ivermectin-sensitive phenotype. In addition, about half of the 52 related cats also included in the present study were heterozygous [MDR1(+/-)] for this MDR1 mutation, while all others were homozygous wild types [MDR1(+/+)]. According to the owners, none of them had a history of drug-sensitivity in case of previous treatments with ivermectin. This observation supports the hypothesis of a strong relationship between a homozygous *ABCB1*1930_1931del TC mutation and an ivermectin-sensitivity.

Interestingly, administered ivermectin doses and severity of neurological signs seen in the two affected Maine Coon cats of the present study are similar to those reported on MDR1 mutant dogs. The female cat (# 1) received an off-label treatment with 0.2 mg/kg (which nevertheless corresponds to the recommended dosage) and showed severe neurological signs for several weeks. Symptoms of the male cat (# 2), which received an off-label treatment with 0.1 mg/kg, were less severe and lasted only for a few days. In dogs with homozygous nt230(del4) MDR1 mutation, mild neurological signs were observed at oral doses of 0.1 mg/kg ivermectin (18, 19). Doses of 0.2–0.6 mg/kg led to severe neurotoxicity with coma and death (10). Subcutaneous injections of 0.2–0.25 mg/kg ivermectin also induced severe neurological signs in MDR1(-/-) dogs, including stupor and coma for several weeks (20). In contrast, MDR1(+/+) dogs tolerate ivermectin at doses up to 2.5 mg/kg without developing neurological signs (5). One cat genotyped by Mealey and Burke (13) as homozygous for the *ABCB1*1930_1931del TC mutation developed progressive CNS depression beginning 6 h after subcutaneous exposure to 0.2 mg/kg ivermectin and lasting for several days (13). Another MDR1(-/-) cat was euthanized after developing severe neurological signs due to unintended oral exposure of up to 523 μg ivermectin/kg (14). Interestingly, there are also some reports of cats recovering completely after administration of much higher doses of ivermectin (21, 22). Moreover, in some cases severity and duration of neurological signs were reported to differ significantly between individual cats despite exposure to the same dosage of macrocyclic lactones (22–24). However, none of these cats were genotyped for the *ABCB1*1930_1931del TC mutation. Therefore, in these cases it can only be speculated that tolerance to overdoses of ivermectin as well as individual variability in drug response is related to different MDR1 genotypes. Nevertheless, other reasons responsible for the observed differences among individual cats, such as differences in body condition scores (22) or age (25), cannot be excluded. According to the owner, the two suspected ivermectin-sensitive Maine Coon cats of the present study were of good general condition. However, in these cases, the cats were treated with a second drug, praziquantel, at the same time. Since praziquantel is known to have a wide margin of safety and low acute toxicity (26, 27), it is less likely that praziquantel contributed to the neurological signs observed in both Maine Coon cats.

Taken together, many factors of the present cases strongly suggest a causal relationship between the homozygous *ABCB1*1930_1931del TC mutation and the suspected ivermectin-sensitivity in both Maine Coon cats. However, further research is needed to finally proof this hypothesis. In this regard, a few additional questions should be addressed. For instance, it remains unclear whether the described 2-bp deletion in the MDR1 gene generates a completely nonfunctional P-gp. Thus, although the mutation leads to a frameshift resulting in an approximately 50 % truncated protein, it cannot be fully excluded if a homodimer with residual activity can be formed from two truncated MDR1 proteins, as it is known from other members of the ABC transporter family (28). To clarify this question, *in vitro* transport studies are needed. Such studies would also be helpful to identify further drugs interacting with the feline MDR1 transporter. Existing literature indicates that cats with homozygous *ABCB1*1930_1931del TC mutation also show increased susceptibility to other P-gp substrates, such as eprinomectin (14). In this regard, a retrospective study analyzing a larger number of adverse event reports on cats exhibiting neurological signs after treatment with P-gp substrates would be useful.

This type of study could also address the question of whether cats with the heterozygous MDR1(+/-) genotype suffer from ivermectin-sensitivity as well. In the present study, none of the MDR1(+/-) cats treated with ivermectin developed any neurological signs. Nevertheless, all of these cats were related to each other. Therefore, it remains unclear whether these results are applicable to other MDR1(+/-) cats and if there is a kind of “intermediate macrocyclic lactone sensitive phenotype” as it is considered for MDR1(+/-) dogs (10). Moreover, a retrospective study could provide information concerning a possible breed predisposition. The current study focused on two suspected ivermectin-sensitive Maine Coon cats and their relatives. Interestingly, in a prospective study, Mealey et al. investigated about 1,000 DNA samples from cats of different breeds. Among them also 20 Maine Coon cats, which however all showed the wild-type MDR1(+/+) genotype (14). To identify predisposed cat breeds and to get a representative estimate about the general allele frequency on the *ABCB1*1930_1931del TC mutation in cats from Europe, a larger number of DNA samples is needed.

The cat-specific TaqMan AD assay developed and validated in the present study provides a useful routine genotyping method for such future studies. The assay has been shown to be a reliable method for the detection of the *ABCB1*1930_1931del TC mutation in DNA samples isolated from both blood and buccal swabs. All genotypes determined by the TaqMan AD assay could be confirmed by DNA sequencing. Since cell material from buccal swabs is considerably less abundant compared to blood samples (29), the DNA amount might be insufficient for TaqMan AD in some cases. Therefore, in the present study, two buccal swabs per animal were *a priori* obtained and investigated per animal, allowing TaqMan AD MDR1 genotyping in almost all cases. Since most of the cats investigated in the current study belonged to the same breeding line, it cannot be excluded that SNPs might be present at the primer and probe hybridization sites of other cats. At least theoretically, SNPs at the primer and probe sites can influence the TaqMan AD. Finally, the TaqMan AD assay described in the present study offers a new method for detecting the feline *ABCB1*1930_1931del TC mutation with high specificity and reliability. This assay can now be applied for routine diagnostic pre-treatment MDR1 genotyping in order to identify drug-sensitive cats and to improve therapeutic safety.

In conclusion, findings of the present study highly suggest a causal relationship between the homozygous *ABCB1*1930_1931del TC [syn. MDR1 nt1930(del2)] mutation in cats and the observed ivermectin-sensitivity. Further studies are needed to confirm this hypothesis and to identify whether affected cats also show increased susceptibility to other P-gp substrates. Moreover, MDR1 genotyping of a larger number of cats is now indicated to get an estimate about the overall allele frequency of the *ABCB1*1930_1931del TC mutation in cats from Europe. The TaqMan AD assay described in the present study provides a useful and reliable method for these purposes. In addition, it is a valuable method for routine MDR1 genotyping to identify drug-sensitive cats prior to treatment with P-gp substrates and to improve therapeutic safety.

## Data Availability Statement

The original contributions presented in the study are included in the article/supplementary material, further inquiries can be directed to the corresponding author.

## Ethics Statement

The animal study was reviewed and approved by Animal Welfare Authorities (Regierungspräsidium Giessen; registration no: 19 c 20 15 h 02 Gi 18/11 kTV 10/2021). Written informed consent was obtained from the owners for the participation of their animals in this study.

## Author Contributions

DN, SL, RL, JA, MH, and JG conceived and designed the project. DN, LW, SM, SL, and JA performed the experiments. DN, SL, JA, MH, and JG analyzed and interpreted the data. DN drafted the first manuscript. DN and SM prepared the figures. DN, MH, and JG critically edited and revised the manuscript. All authors contributed to the article and approved the final version of the manuscript.

## Funding

Funding for this study was received from the Bundesamt für Verbraucherschutz und Lebensmittelsicherheit (BVL, Braunschweig/Berlin, Germany) *via* the pharmacovigilance program.

## Conflict of Interest

The authors declare that nt230(del4) MDR1 genotyping in dogs is a for-profit licensed and patent-protected diagnostic service of their host institute offered via TransMIT GmbH Center for Pharmacogenetic Diagnostics (PGvet). Joachim Geyer is head of PGvet. The authors declare that the research was conducted in the absence of any commercial or financial relationships that could be construed as a potential conflict of interest.

## Publisher's Note

All claims expressed in this article are solely those of the authors and do not necessarily represent those of their affiliated organizations, or those of the publisher, the editors and the reviewers. Any product that may be evaluated in this article, or claim that may be made by its manufacturer, is not guaranteed or endorsed by the publisher.

## Abbreviations

ABCB1: ATP-binding cassette subfamily B member 1
AD: Allelic discrimination
CNS: Central nervous system
MDR1
Multidrug resistance gene
MGB: Minor groove binder
NFQ: Non-fluorescent quencher
NTC: No-template control
P-gp: P-glycoprotein
SNP: Single nucleotide polymorphism.
